# A guide to aid the selection of diagnostic tests

**DOI:** 10.2471/BLT.16.187468

**Published:** 2017-06-26

**Authors:** Cara S Kosack, Anne-Laure Page, Paul R Klatser

**Affiliations:** a Médecins Sans Frontières, Plantage Middenlaan 14, 1018 DD Amsterdam, Netherlands.; b Epicentre, Paris, France.; c Academic Medical Center, Amsterdam, Netherlands.

## Abstract

In recent years, a wide range of diagnostic tests has become available for use in resource-constrained settings. Accordingly, a huge number of guidelines, performance evaluations and implementation reports have been produced. However, this wealth of information is unstructured and of uneven quality, which has made it difficult for end-users, such as clinics, laboratories and health ministries, to determine which test would be best for improving clinical care and patient outcomes in a specific context. This paper outlines a six-step guide to the selection and implementation of in vitro diagnostic tests based on Médecins Sans Frontières’ practical experience: (i) define the test’s purpose; (ii) review the market; (iii) ascertain regulatory approval; (iv) determine the test’s diagnostic accuracy under ideal conditions; (v) determine the test’s diagnostic accuracy in clinical practice; and (vi) monitor the test’s performance in routine use. Gaps in the information needed to complete these six steps and gaps in regulatory systems are highlighted. Finally, ways of improving the quality of diagnostic tests are suggested, such as establishing a model list of essential diagnostics, establishing a repository of information on the design of diagnostic studies and improving quality control and postmarketing surveillance.

## Résumé

Depuis quelques années, de multiples tests diagnostiques sont disponibles dans les lieux de soins disposant de ressources limitées. En conséquence, un nombre considérable de directives, d'évaluations des performances et de rapports de mise en œuvre ont été élaborés. Cette masse d'informations manque néanmoins de structure et est de qualité inégale, raisons pour lesquelles les utilisateurs finaux, tels que les centres de santé, les laboratoires et les ministères de la Santé, ont eu des difficultés à déterminer quel test conviendrait le mieux pour améliorer les soins cliniques et l'état de santé des patients dans un contexte donné. Ce document présente un guide en six étapes pour faciliter la sélection et la mise en œuvre de tests diagnostiques in vitro, en s'appuyant sur l'expérience pratique de Médecins Sans Frontières: (i) définir l'objectif du test; (ii) analyser le marché; (iii) obtenir l'approbation réglementaire; (iv) déterminer la précision du test diagnostique dans des conditions idéales; (v) déterminer la précision du test diagnostique dans le cadre d'une pratique clinique; et (vi) suivre les performances du test dans le cadre d'une utilisation courante. Ce document met en avant les informations manquantes nécessaires pour accomplir ces six étapes et les lacunes des systèmes de réglementation. Enfin, il propose des moyens d'améliorer la qualité des tests diagnostiques, à travers l'établissement d'une liste modèle des diagnostics essentiels, l'élaboration d'un référentiel d'informations sur la réalisation des études diagnostiques, et l'amélioration du contrôle de la qualité et de la pharmacovigilance.

## Resumen

En los últimos años se ha desarrollado una amplia variedad de pruebas de diagnóstico para el uso en entornos con recursos limitados. Como consecuencia, se ha producido una gran cantidad de manuales, evaluaciones de rendimiento e informes de implementación. No obstante, esta abundancia de información está desestructurada y tiene una calidad irregular. Esto ha dificultado que los usuarios finales, como clínicas, laboratorios y ministerios de salud, puedan determinar qué prueba es la más indicada para mejorar la atención sanitaria y los resultados de los pacientes en un contexto específico. En el presente informe se describe un manual de seis pasos para la selección y la implementación de pruebas de diagnóstico in vitro sobre la base de la experiencia práctica de Mèdecins Sans Frontières: (i) definir el propósito de la prueba; (ii) revisar el mercado; (iii) verificar la aprobación normativa; (iv) determinar la precisión del diagnóstico de la prueba en condiciones ideales; (v) determinar la precisión del diagnóstico de la prueba en la práctica clínica; y (vi) controlar el rendimiento de la prueba en su uso habitual. Se destacan los vacíos en la información necesarios para completar estos seis pasos y los vacíos del sistema normativo. Finalmente, se sugieren maneras para mejorar la calidad de las pruebas de diagnóstico, como establecer una lista modelo de los diagnósticos esenciales, establecer un repositorio de información sobre el diseño de los estudios de diagnósticos y mejorar el control de calidad y el control del postmarketing.

## ملخص

توافرت في السنوات الأخيرة مجموعة واسعة من الاختبارات التشخيصية لاستخدامها في البيئات شحيحة الموارد. ولذلك، تم وضع عدد هائل من المبادئ التوجيهية وتقييمات الأداء وتقارير التنفيذ. ومع ذلك، فإن ثروة المعلومات هذه تظل غير منظمة وغير متكافئة الجودة، مما جعل من الصعب على المستخدمين النهائيين، مثل العيادات والمختبرات ووزارات الصحة، تحديد أي اختبار سيكون أفضل لتحسين الرعاية السريرية ونتائج المرضى في سياق محدد. وتحدد هذه الدراسة دليلاً من ست خطوات لانتقاء الاختبارات التشخيصية المختبرية وتنفيذها استنادًا إلى الخبرة العملية لمنظمة أطباء بلا حدود: (أ) تحديد الغرض من الاختبار؛ (ب) مراجعة السوق؛ (جـ) التحقق من الموافقة التنظيمية؛ (د) تحديد الدقة التشخيصية للاختبار في ظل ظروف مثالية؛ (هـ) تحديد دقة تشخيص الاختبار في الممارسة السريرية؛ (و) مراقبة أداء الاختبار في الاستخدام الروتيني. وتم تسليط الضوء على الثغرات الموجودة في المعلومات اللازمة لاستكمال هذه الخطوات الست والثغرات الموجودة في النظم التنظيمية. وأخيرًا، تم اقتراح طرق لتحسين نوعية الاختبارات التشخيصية، مثل إنشاء قائمة نموذجية من التشخيصات الأساسية، وإنشاء مستودع للمعلومات حول تصميم الدراسات التشخيصية، وتحسين مراقبة الجودة، ومراقبة ما بعد التسويق.

## 摘要

近年来，各种各样的诊断测试可在资源有限的环境中使用。 相应地，形成了大量指南、表现评估和实施报告。 然而，这些丰富的信息是松散的、质量参差不齐，使诊所、实验室和卫生部这样的终端用户难以决定哪一种测试在具体环境下最适合改善临床护理和患者效果。 本文概述了基于 Médecins Sans Frontières 的实践经验的六步骤体外诊断测试选择和实施指南： (i) 定义测试目的；(ii) 回顾市场；(iii) 确定监管部门的批准；(iv) 在理想条件下确定测试的诊断精度； (v) 在临床实践中确定测试的诊断精度；和 (vi) 在日常使用中监控测试性能。 信息中的差距需要完成这六个步骤，突出了监管体系中的差距。 最后，提出了提高诊断测试质量的方法，例如建立关键诊断的模型列表、建立诊断研究设计信息资源库以及改善质量控制和售后监督。

## Резюме

В последние годы широкий диапазон диагностических тестов стал доступен для использования в условиях ограниченных ресурсов. В связи с этим было подготовлено огромное количество руководств, оценок эффективности и отчетов о реализации. Однако это изобилие информации не систематизировано и имеет неоднородное качество, что затрудняет конечным пользователям, таким как клиники, лаборатории и министерства здравоохранения, определять, какой тест лучше всего подходит для улучшения лечебной работы и результатов лечения пациентов в конкретном контексте. В этом документе описывается шестиэтапное руководство по выбору и внедрению диагностических тестов in vitro на основе практического опыта организации «Врачи без границ» (Médecins Sans Frontières): (i)  определить цель испытания; (ii)  провести обзор рынка; (iii)  убедиться в наличии официального утверждения; (iv)  определить точность диагностики теста в идеальных условиях; (v)  определить точность диагностики теста в клинической практике; и (vi)  контролировать проведение теста при обычном использовании. Сделан акцент на пробелах в информации, необходимой для выполнения этих шести этапов, и пробелах в системах регулирования. Наконец, предлагаются пути повышения качества диагностических тестов, такие как составление перечня основных диагностических средств, создание хранилища информации о разработке диагностических исследований и совершенствование контроля качества и постмаркетингового надзора.

## Introduction

Diagnostic testing has become indispensable for diagnosing and monitoring disease, for providing prognoses and for predicting treatment responses.^1^^,^^2^ Today, over 40 000 products are available globally for the in vitro diagnostic testing of a wide range of conditions.^3^ These include traditional laboratory-based tests, with samples being sent to a central laboratory for analysis, and point-of-care tests, which can be performed near, or at, the point of patient care. Point-of-care testing can help optimize treatment decision-making, avoid referrals, improve the efficiency of care and decrease costs, especially in resource-constrained settings where laboratory infrastructure is weak.^4^

In the early 1990s, the first point-of-care tests for use in resource-constrained settings became commercially available: lateral flow immunoassays (often called rapid diagnostic tests) for the diagnosis of malaria.^5^^,^^6^ These assays are now well established and have replaced blood film microscopy in many settings. However, as the market for diagnostic tests has increased, the choice has become overwhelming for some diseases: in 2015, the World Health Organization (WHO), the Foundation for Innovative New Diagnostics (FIND) and the Centers for Disease Control and Prevention (CDC) in the United States of America reviewed approximately 250 different diagnostic tests for malaria.^7^ We conducted an online market search for screening tests for hepatitis C virus infection and identified more than 50 products, and UNITAID’s 2015 report on tuberculosis diagnostics highlighted the increasing complexity of the market,^8^ with WHO endorsing (though not prequalifying) several products in recent years.^9^^–^^12^ In some cases, a single manufacturer may hold a monopoly, which can lead to high costs. Conversely, diagnostic and monitoring tests for neglected diseases, such as visceral leishmaniasis, human African trypanosomiasis, chikungunya, dengue and brucellosis, remain scarce.^13^^,^^14^

With the increase in the number of in vitro diagnostic tests has come an increase in the number of guidelines and recommendations, together with countless publications on their performance and implementation.^7^^,^^13^^,^^15^^–^^17^ However, this wealth of material covers only a small proportion of commercially available tests. In our experience, the information available on many tests is limited and there is often a lack of independent data on a test’s performance and on whether the manufacturing process is reliable enough to ensure consistent quality across multiple lots. Both the quantity and the variable quality of the information available make it difficult for policy-makers, laboratories and other end-users to make rational decisions about the selection and use of these tests.^18^^,^^19^ As a result, tests have been used unnecessarily and incorrectly and results have been misinterpreted.^20^^–^^23^

Given these difficulties, the process of selecting one or several tests for use in a diagnostic algorithm can be cumbersome for clinics, countries and nongovernmental organizations providing medical support.^19^ The nongovernmental organization, Médecins Sans Frontières, operates or supports health ministry laboratories in more than 40 countries. Currently, the organization has over 15 laboratory advisors at its headquarters and more than 100 staff working in laboratories around the world. The amount of laboratory equipment and the number of in vitro diagnostic tests used by Médecins Sans Frontières itself have almost doubled in the past 10 years and the organization has encountered numerous challenges in selecting and implementing tests for its projects.

In this article, we outline a six-step approach to overcoming the obstacles encountered by Médecins Sans Frontières in selecting and implementing in vitro diagnostic tests. This approach was derived from a review of the diagnostics literature and from our experience with implementing diagnostics programmes. We discuss the challenges involved in each of the six steps and outline current problems with the quality assurance of tests. We hope this simple stepwise guide will help clinics, organizations and health ministries to make rational decisions about the selection of in vitro diagnostic tests and, over the longer term, will contribute to the development of a practical guide for selecting diagnostics.

## Selecting a test

The ASSURED (Affordable, Sensitive, Specific, User-friendly, Rapid and robust, Equipment-free and Deliverable to end-users) criteria can be used as a benchmark for identifying the most appropriate diagnostic tests for resource-constrained settings.^24^ However, these criteria are generic and need to be adapted to each diagnostic need. In addition, not all test methods can be simplified to fit the ASSURED criteria: for instance, both laboratory infrastructure and equipment are required for diagnosing human immunodeficiency virus (HIV) infections in young infants using dried blood spot collection and for monitoring HIV viral load in infected individuals. In these situations, the test’s specification must be broadened during the selection process.

We have identified six steps that must be addressed when selecting an in vitro diagnostic test: (i) define the test’s purpose; (ii) review the market and check each product’s specification; (iii) review the test’s regulatory approval; (iv) obtain data on the diagnostic accuracy of the test under ideal conditions (i.e. in laboratory-based evaluations); (v) obtain data on the diagnostic accuracy of the test in clinical practice; and (vi) monitor the test’s performance in routine use (Box 1). These six steps, along with context-specific barriers to use, should all be considered when selecting an in vitro diagnostic test for routine use.

Box 1 **Steps in selecting a diagnostic test**

**Step 1: Define the test’s purpose – why, what, where, who?**

- Decide whether an acute or chronic infection is to be diagnosed
- Decide whether the test is to be used for diagnosis, disease monitoring or verifying a cure
- Decide whether the test should be quantitative or qualitative
- Decide whether test results will be analysed at the point of care or in a central laboratory
- Define the test’s end-users: trained laboratory technicians or primary health-care workers?
- What is the required performance of the test?

**Step 2: Review the market**

- Identify the products of interest available
- Obtain details of the tests available, including: (i) the manufacturer’s name; (ii) the product’s name; (iii) the product’s catalogue number; (iv) package size; (v) storage requirements; (vi) shelf life; (vii) sample type (e.g. serum, plasma, whole blood or urine) and volume required; (viii) control reagents available; (ix) instruction languages; (x) how long the test takes and the number of steps required; (xi) additional equipment required; and (xii) cost
- Determine whether analysers are used and, if so, what the manufacturer’s requirements are for training, installation and maintenance

**Step 3: Review regulatory approval by international and national bodies**

- Determine whether the test has the European CE mark
- Determine whether the test has been approved by the FDA
- Determine whether the test’s manufacturing site meets the ISO 13485 standard
- Determine whether the test is prequalified or endorsed by WHO
- If not prequalified or endorsed by WHO, determine whether the test has been approved by the Expert Review Panel of the Global Fund to Fight AIDS, Tuberculosis and Malaria
- Determine whether the test has been approved by national authorities

**Step 4: Determine the test’s optimal diagnostic accuracy**

- Review publications on the test’s performance under ideal conditions (i.e. at reference laboratories)

**Step 5: Determine the test’s diagnostic accuracy in practice**

- Review publications on the test’s performance under real-life conditions (i.e. at the end-user level)

**Step 6: Monitor the test in routine use**

- Carry out quality control
- Carry out proficiency testing
- Supervise and train end-users

CE: Conformité Européene; FDA: Food and Drug Administration; ISO: International Organization for Standardization; WHO: World Health Organization.

### Step 1: Defining the test

Clearly defining the test’s purpose is important, because this will influence many of the subsequent steps in the selection process. Factors to be considered include: (i) the disease or condition to be diagnosed; (ii) whether a single test or a diagnostic algorithm is required; and (iii) whether the test should, or can, provide a qualitative or quantitative result. In addition, for optimal clinical utility, consideration should be given to: (i) the site of testing (e.g. in a large laboratory or a small health-care centre); and (ii) the end-user (e.g. a well-trained laboratory technician or a primary health-care worker, such as a nursing assistant). Other important considerations are the clinical use of the test (e.g. whether a screening or confirmatory test is required) and the added value of the test or combination of tests.^25^^,^^26^

### Step 2: Market review

The market should be reviewed to identify the tests available for the condition of interest by consulting guidance from international organizations such as WHO and manufacturers’ product information. Product specifications may include details of the type of sample required, the test’s operating conditions, additional equipment required and shelf life. The veracity of manufacturers’ claims should be judged by searching the peer-reviewed medical literature. The manufacturer often overstates a test’s diagnostic accuracy in test brochures or instructions for use: for example, its sensitivity and specificity may not have been replicated in independent evaluations.^27^ Unfortunately, few prosecutions for inappropriate claims have been pursued by national authorities. The selection process can also be hindered by local market problems with counterfeit tests and by a lack of regulation.^4^^,^^28^^–^^30^

### Step 3: Regulatory approval

Many countries do not have regulatory procedures in place for assessing the safety, quality or effectiveness of in vitro diagnostic tests, which means that poor-quality tests can be marketed and used.^4^^,^^28^^,^^30^ However, test selection can be assisted by consulting recommendations from international regulatory bodies, for some major conditions at least. For example, WHO has established a prequalification process for in vitro diagnostic tests for diseases with a high individual or public health risk – the process has a particular focus on ensuring that tests for HIV, malaria and hepatitis B and C infections are affordable in resource-constrained settings.^31^ The prequalification process has three main components: (i) a review of the product application and dossier; (ii) laboratory evaluation of the product; and (iii) inspection of the manufacturing site. The process thus assesses both the test’s performance and manufacturing quality. More than 60 products have been prequalified since the process started in 2010.^13^ Recently, tests for glucose-6-phosphate dehydrogenase deficiency, screening for human papillomavirus and emergency assessments in the outbreak of diseases such as Ebola and Zika have been included, but many others have not.

Similarly, the Global Fund to Fight AIDS, Tuberculosis and Malaria has produced a list of in vitro diagnostic tests eligible for procurement, which is based on WHO’s recommendations and on products approved by the regulatory authorities of the founding members of the Global Harmonization Task Force: Australia, Canada, the European Union, Japan and the United States.^32^ Other authorities and donors may use different lists. For example, the President's Emergency Plan For AIDS Relief (PEPFAR) relies on the United States Agency for International Development’s list of approved tests for HIV/AIDS.^33^ Although these sources list a broad range of recommended products, guidance on the use of diagnostic tests for conditions other than HIV infection, malaria and tuberculosis is scarce. Moreover, few in vitro diagnostic tests for neglected diseases or products for monitoring the side-effects of drug therapy have been endorsed, or approved for use, by international agencies.

### Step 4: Optimal diagnostic accuracy

The performance of a test under ideal conditions (i.e. in phase-II studies) indicates its optimal performance. This information is crucial for enabling users to preselect a test for a trial under real-life conditions. Such evaluations may provide important information not only on a test’s diagnostic accuracy but also on its repeatability, reproducibility and ease of use and on variations between production lots. For some infectious diseases, evaluations of diagnostic tests are carried out at regular intervals by international stakeholders and the results are made publicly available: for example, tests for malaria are routinely evaluated by WHO and FIND and other tests are monitored during WHO’s prequalification process.^7^ No similar evaluations have been carried out for many other conditions. Nevertheless, guidance on the evaluation of diagnostic tests for several infectious diseases has been provided in several publications.^34^^,^^35^ Individual organizations may find it difficult to carry out these evaluations themselves because often they can be performed only by reference laboratories with high-quality infrastructure and highly skilled-staff.

Specimen banks can provide material for evaluating in vitro diagnostic tests and can be useful for helping manufacturers develop high-quality diagnostic products.^36^ Unfortunately, we only know of specimen banks for tuberculosis and human African trypanosomiasis. In addition, WHO, FIND and the CDC provide malaria specimens for manufacturers evaluating prototypes of rapid diagnostic tests. Access to specimen banks, especially for neglected infectious diseases, would be hugely beneficial for the development and monitoring of in vitro diagnostic tests.

### Step 5: Diagnostic accuracy in practice

Both the actual performance of in vitro diagnostic tests and their ease of use should also be considered during the selection process. Evaluations at the end-user level (i.e. phase-III studies) provide information on a test’s performance under real-life conditions and can reveal important features that were not revealed in phase-II studies. In practice, a test’s performance in the field can be influenced by the user’s level of training and by environmental conditions, such as a high temperature or humidity and dust. Evaluations under real-life conditions are essential because often staff carrying out diagnostic testing in resource-constrained settings (e.g. HIV counsellors) will not have had the same training as laboratory workers at reference laboratories. Also, an end-user’s perception of a test’s ease of use may be quite different from that of staff at a reference laboratory. Real-life evaluations should be conducted in the population in which the test will be used as this will provide clinical accuracy data that are appropriate for the prevalence of the disease locally and for other context-specific factors that could influence accuracy, such as common comorbidities.

If no phase-III evaluations have been carried out, end-users should consider performing such evaluations themselves. In practice, national regulatory authorities often require phase-III studies to be performed before approving the introduction of an in vitro diagnostic test. Such evaluations can be conducted by the health ministry, test developers or other actors. Some guidance exists: for example, guidelines from WHO, CDC and the Association of Public Health Laboratories on the evaluation of HIV testing technologies in Africa^37^ and generic protocols developed by the International Diagnostics Centre.^38^ Again, these focus on only a few diseases.

The results of diagnostic accuracy studies can be obtained from public reports or from the peer-reviewed literature. When reviewing publications, it is important to be aware of the stage at which the test was evaluated, which could be: (i) a prototype evaluation (phase I); (ii) an evaluation under ideal conditions (phase II); or (iii) an evaluation under real-life conditions (phase III). Next, the quality of the study data should be assessed. The Standards for Reporting Diagnostic Accuracy (STARD), updated in 2015, provide guidance on improving the quality of reporting of research on diagnostic test accuracy but, unfortunately, much reporting is still incomplete.^39^ Nevertheless, these standards can help end-users judge the quality of the study being assessed. It should also be noted that reviewing the literature on diagnostic tests can provide useful information on the challenges faced when introducing and routinely using new tests. Recently, the Cochrane Collaboration has started conducting systematic reviews and meta-analyses of diagnostic test accuracy studies.^40^ These reviews are comprehensive, provide information on the quality and reliability of studies, and can greatly help end-users interpret published data.

### Step 6: Monitoring performance

Monitoring a test’s performance in routine use is important. Quality control, proficiency testing and the supervision of end-users should be carried out regularly and documented. Postmarketing surveillance is another important component of long-term quality assurance. This is usually performed by the relevant national authorities and is included in WHO’s prequalification process, to which end-users can contribute. Postmarketing surveillance is both reactive (e.g. in response to complaints by procurers and end-users) and proactive (e.g. in verifying the quality of production lots both before and after distribution). In addition, end-users’ reports about prequalified tests are collected and investigated during WHO’s prequalification process. Unfortunately, neither end-users’ complaints nor analyses of these complaints are publicly available. However, WHO does publish field safety notices if complaints are substantiated.

### Gaps in guidance

The diagnostic accuracy of in vitro diagnostic tests has been evaluated mainly in diseases subject to major control efforts, such as HIV infection, tuberculosis and malaria. However, even for these diseases, no comprehensive, structured, pragmatic guidelines exist that can be used to help national governments, diagnostic programmes or laboratories with selecting tests. The absence of guidance is an even greater problem when diagnosis requires more than a simple rapid test. Guidance on how to choose, implement or monitor more complex diagnostic methods is also scarce. Furthermore, procurement guides are nonexistent, with two notable exceptions: WHO’s procurement guides on laboratory equipment for HIV testing and on rapid diagnostic tests for malaria.^41^^,^^42^

Finally, most existing guidelines do not fully consider cost. This is particularly important for more complex tests because the cost of transport, storage, remodelling laboratory structures, training and supply chain management, for example, need to be considered together with the cost of the test itself.^43^ According to WHO’s CHOICE (Choosing Interventions that are Cost-Effective) project,^44^ cost–effectiveness studies that use thresholds based on per-capita income provide little guidance because they disregard budgetary constraints. For example, it has been estimated that, although use of the GeneXpert MTB/RIF test in 15% of suspected tuberculosis cases in India would be cost-effective at 2010 prices, the test would consume the entire budget of the country’s tuberculosis programme.^45^ However, alternative ways of evaluating cost–effectiveness in low- and middle-income countries have been proposed and could be incorporated into the steps outlined here.^46^

## The way forward

We hope this stepwise guide will help stakeholders select, implement and monitor in vitro diagnostic tests. Each of the steps outlined should be elaborated into practical guidelines. The comprehensive and accessible online laboratory quality stepwise implementation tool from WHO,^47^ which provides medical laboratories with a guide to implementing quality management systems in compliance with ISO (International Organization for Standardization) 15189 standards, may serve as a model.

There have been calls for a model list of essential diagnostics comparable with the *Model list of essential medicines* maintained by WHO.^48^^–^^50^ The idea was first proposed by WHO in January 2017.^51^ Such a list would help in the selection of diagnostic methods and would facilitate improvements in the regulation and affordability of in vitro diagnostic tests and in training in their use. The list should be based on the prevalence, and the relevance to public health, of the diseases considered and not limited to only a few diseases, as are some other regulatory processes. Another challenge is to find the right way of specifying diagnostic tests because several tests might have the same generic name but be very different in terms of quality and performance. Although a model list of essential diagnostics would provide information on diagnostic requirements and test characteristics, it will still be necessary to establish selection criteria for individual tests.

The quality of test evaluations and reports could be improved by establishing a repository of information on, or a central point of assistance for, the design of diagnostic studies. In Africa, the Collaboration for Evidence-Based Health Care in Africa or the African Society for Laboratory Medicine could serve this function. The protocols used for evaluations carried out during WHO’s prequalification process should be made publicly available along with information on how other diagnostic test evaluations are taken into account. In addition, peer-reviewed journals should be more rigorous in checking whether STARD criteria have been followed in studies submitted for publication.

Diagnostic tests must be manufactured under strict conditions to ensure their consistent performance. Consistency is crucial and its verification should be taken into account during test selection. Moreover, production lots could be tested independently, as has been proposed for malaria tests by WHO and FIND.^52^ Improvements are also needed in the continuous monitoring of test quality and in feedback to end-users. An initial step could be for each laboratory and test centre to have access to internal and external quality controls. Currently, few manufacturers have made positive and negative control materials commercially available for evaluating rapid diagnostic tests and few laboratories or test centres are involved in proficiency testing. Access to information on postmarketing surveillance also needs to be improved; in addition to field safety notices, the results of lot testing and information on complaints about tests should also be made publicly available.

With the guidance presented here and implementation of the improvements suggested, diagnostic testing will have a greater chance of realizing its potential for improving patient care in resource-constrained settings.

## References

[1] Raman R, Avendano EE, Chen M. Update on emerging genetic tests currently available for clinical use in common cancers. Technology assessment report. Tufts Evidence-based Practice Center. Rockville: Agency for Healthcare Research and Quality; 2013. Available from: https://www.ncbi.nlm.nih.gov/pubmedhealth/PMH0073338/pdf/PubMedHealth_PMH0073338.pdf [cited 2017 June 9]. 25905153

[2] Billings PR. Three barriers to innovative diagnostics. Nat Biotechnol. 2006 8;24(8):917–8. 10.1038/nbt0806-917 16900129

[3] Rohr U-P, Binder C, Dieterle T, Giusti F, Messina CG, Toerien E, et al. The value of in vitro diagnostic testing in medical practice: a status report. PLoS One. 2016 3 4;11(3):e0149856. 10.1371/journal.pone.0149856 26942417 PMC4778800

[4] Mori M, Ravinetto R, Jacobs J. Quality of medical devices and in vitro diagnostics in resource-limited settings. Trop Med Int Health. 2011 11;16(11):1439–49. 10.1111/j.1365-3156.2011.02852.x 21955331

[5] Cavallo JD, Hernandez E, Gerome P, Plotton N, Debord T, Le Vagueresse R. Antigénémie HRP-2 et paludisme d’importation à Plasmodium falciparum: comparaison du ParaSight-F et de l’ICT malaria P.f. Med Trop (Mars). 1997;57(4):353–6 (in French). 9612775

[6] Shiff CJ, Premji Z, Minjas JN. The rapid manual ParaSight-F test. A new diagnostic tool for Plasmodium falciparum infection. Trans R Soc Trop Med Hyg. 1993 Nov-Dec;87(6):646–8. 10.1016/0035-9203(93)90273-S 8296363

[7] Malaria rapid diagnostic test performance. Summary results of WHO product testing of malaria RDTs: rounds 1–6 (2008–2015). Geneva: World Health Organization; 2015. Available from: http://apps.who.int/iris/bitstream/10665/204119/1/9789241510042_eng.pdf?ua=1 [cited 2017 June 9].

[8] Tuberculosis. Diagnostics technology and market landscape. 4th edition. Vernier: UNITAID; 2015. Available from: https://www.unitaid.eu/assets/Tuberculosis_diagnostics_technology_and_market_landscape_4th_edition_Oct_2015-1.pdf [cited 2017 June 9].

[9] The use of molecular line probe assays for the detection of resistance to isoniazid and rifampicin. Policy update. Geneva: World Health Organization; 2016. Available from: http://apps.who.int/iris/bitstream/10665/250586/1/9789241511261-eng.pdf?ua=1 [cited 2017 June 9].

[10] The use of molecular line probe assays for the detection of resistance to second-line anti-tuberculosis drugs: policy guidance. Geneva: World Health Organization; 2016. Available from: http://www.who.int/tb/WHOPolicyStatementSLLPA.pdf [cited 2017 June 9].

[11] The use of lateral flow urine lipoarabinomannan assay (LF-LAM) for the diagnosis and screening of active tuberculosis in people living with HIV. Policy guidance. Geneva: World Health Organization; 2015. Available from: http://www.who.int/tb/areas-of-work/laboratory/policy_statement_lam_web.pdf [cited 2017 June 9].

[12] Automated real-time nucleic acid amplification technology for rapid and simultaneous detection of tuberculosis and rifampicin resistance: Xpert MTB/RIF assay for the diagnosis of pulmonary and extrapulmonary TB in adults and children: policy update. Geneva: World Health Organization; 2013. Available from: http://apps.who.int/iris/bitstream/10665/112472/1/9789241506335_eng.pdf [cited 2017 June 9]. 25473701

[13] WHO list of prequalified in vitro diagnostic products. Last update: 14 July 2016. Geneva: World Health Organization; 2016. Available from: http://www.who.int/diagnostics_laboratory/evaluations/160714_prequalified_product_list.pdf?ua=1 [cited 2017 June 9].

[14] Visceral leishmaniasis rapid diagnostic test performance. Diagnostics evaluation series no. 4. Geneva: World Health Organization; 2011. p. 46. Available from: http://www.who.int/tdr/publications/documents/vl-rdt-evaluation.pdf [cited 2017 June 9].

[15] Otani MM, Vinelli E, Kirchhoff LV, del Pozo A, Sands A, Vercauteren G, et al. WHO comparative evaluation of serologic assays for Chagas disease. Transfusion. 2009 6;49(6):1076–82. 10.1111/j.1537-2995.2009.02107.x 19290995

[16] Boelaert M, Rijal S, Regmi S, Singh R, Karki B, Jacquet D, et al. A comparative study of the effectiveness of diagnostic tests for visceral leishmaniasis. Am J Trop Med Hyg. 2004 1;70(1):72–7. 14971701

[17] Boehme CC, Nicol MP, Nabeta P, Michael JS, Gotuzzo E, Tahirli R, et al. Feasibility, diagnostic accuracy, and effectiveness of decentralised use of the Xpert MTB/RIF test for diagnosis of tuberculosis and multidrug resistance: a multicentre implementation study. Lancet. 2011 4 30;377(9776):1495–505. 10.1016/S0140-6736(11)60438-8 21507477 PMC3085933

[18] McNerney R. Diagnostics for developing countries. Diagnostics (Basel). 2015 5 19;5(2):200–9. 10.3390/diagnostics5020200 26854149 PMC4665590

[19] Kosack CS. Experience of Médecins Sans Frontières in laboratory medicine in resource-limited settings. Clin Chem Lab Med. 2012 7;50(7):1221–7. 10.1515/cclm-2011-0618 23024983

[20] Asiimwe C, Kyabayinze DJ, Kyalisiima Z, Nabakooza J, Bajabaite M, Counihan H, et al. Early experiences on the feasibility, acceptability, and use of malaria rapid diagnostic tests at peripheral health centres in Uganda–insights into some barriers and facilitators. Implement Sci. 2012 1 23;7(1):5. 10.1186/1748-5908-7-5 22269037 PMC3398266

[21] Heunis JC, Wouters E, Norton WE, Engelbrecht MC, Kigozi NG, Sharma A, et al. Patient- and delivery-level factors related to acceptance of HIV counseling and testing services among tuberculosis patients in South Africa: a qualitative study with community health workers and program managers. Implement Sci. 2011 3 23;6(1):27. 10.1186/1748-5908-6-27 21426586 PMC3074565

[22] Jarosławski S, Pai M. Why are inaccurate tuberculosis serological tests widely used in the Indian private healthcare sector? A root-cause analysis. J Epidemiol Glob Health. 2012 3;2(1):39–50. 10.1016/j.jegh.2011.12.001 23856397 PMC7320362

[23] Grenier J, Pinto L, Nair D, Steingart K, Dowdy D, Ramsay A, et al. Widespread use of serological tests for tuberculosis: data from 22 high-burden countries. Eur Respir J. 2012 2;39(2):502–5. 10.1183/09031936.00070611 22298618

[24] Peeling RW, Holmes KK, Mabey D, Ronald A. Rapid tests for sexually transmitted infections (STIs): the way forward. Sex Transm Infect. 2006 12;82 Suppl 5:v1–6. 10.1136/sti.2006.024265 17151023 PMC2563912

[25] Bossuyt PMM, Reitsma JB, Linnet K, Moons KGM. Beyond diagnostic accuracy: the clinical utility of diagnostic tests. Clin Chem. 2012 12;58(12):1636–43. 10.1373/clinchem.2012.182576 22730450

[26] Moons KGM, de Groot JA, Linnet K, Reitsma JB, Bossuyt PM. Quantifying the added value of a diagnostic test or marker. Clin Chem. 2012 10;58(10):1408–17. 10.1373/clinchem.2012.182550 22952348

[27] Hunsperger EA, Yoksan S, Buchy P, Nguyen VC, Sekaran SD, Enria DA, et al. Evaluation of commercially available anti-dengue virus immunoglobulin M tests. Emerg Infect Dis. 2009 3;15(3):436–40. 10.3201/eid1503.080923 19239758 PMC2681117

[28] Roche position on counterfeiting. Basel: Roche; 2016. Available from: http://www.roche.com/dam/jcr:d1fed4b2-1d2d-4f40-98ff-c5fae6be0ba1/07_Position_Counterfeiting_reviewed_4_2017_inkl.%20FAQ_Update_2017.pdf [cited 2017 June 9].

[29] McNerney R, Peeling RW. Regulatory in vitro diagnostics landscape in Africa: update on regional activities. Clin Infect Dis. 2015 10 15;61(61) Suppl 3:S135–40. 10.1093/cid/civ553 26409274

[30] Rugera SP, McNerney R, Poon AK, Akimana G, Mariki RF, Kajumbula H, et al. Regulation of medical diagnostics and medical devices in the East African community partner states. BMC Health Serv Res. 2014 10 31;14(1):524. 10.1186/s12913-014-0524-2 25366990 PMC4221680

[31] Overview of the prequalification of in vitro diagnostics assessment. WHO prequalification of in vitro diagnostics programme. Version 5. 30 May 2014. Geneva: World Health Organization; 2014. Available from: http://www.who.int/diagnostics_laboratory/evaluations/140530_pqdx_overview_doc_007.pdf?ua=1 [cited 2017 June 9].

[32] List of HIV diagnostic test kits and equipments classified according to the Global Fund quality assurance policy. Version 14. 2017 March 31. Vernier: The Global Fund; 2016. Available from: https://www.theglobalfund.org/media/5878/psm_productshiv-who_list_en.pdf [cited 2017 June 9].

[33] USAID list of approved HIV/AIDS rapid test kits. Washington (DC): USAID; 2015. Available from: https://www.usaid.gov/sites/default/files/documents/1864/approved-rapid-hiv-aids-test-kits-october-2015.pdf [cited 2017 June 9].

[34] Herring A, Ballard R, Mabey D, Peeling RW; WHO/TDR Sexually Transmitted Diseases Diagnostics Initiative. Evaluation of rapid diagnostic tests: syphilis. Nat Rev Microbiol. 2006 12;4(12) Suppl:S33–40. 10.1038/nrmicro1563 17366685

[35] Herring A, Ballard R, Mabey D, Peeling RW; WHO/TDR Sexually Transmitted Diseases Diagnostics Initiative. Evaluation of rapid diagnostic tests: chlamydia and gonorrhoea. Nat Rev Microbiol. 2006 12;4(12) Suppl:S41–8. 10.1038/nrmicro1562 17366686

[36] Vincent V, Rigouts L, Nduwamahoro E, Holmes B, Cunningham J, Guillerm M, et al. The TDR Tuberculosis Strain Bank: a resource for basic science, tool development and diagnostic services. Int J Tuberc Lung Dis. 2012 1;16(1):24–31. 10.5588/ijtld.11.0223 22236841

[37] World Health Organization Regional Office for Africa, Centers for Disease Control and Prevention and Association of Public Health Laboratories. Guidelines for appropriate evaluations of HIV testing technologies in Africa. Geneva: World Health Organization; 2003. Available from: http://www.who.int/hiv/pub/vct/testing_africa/en/ [cited 2017 June 9].

[38] International Diagnostics Centre [Internet]. London: International Diagnostics Centre; 2017. Available from: http://www.idc-dx.org/ [cited 2017 June 9].

[39] Korevaar DA, van Enst WA, Spijker R, Bossuyt PMM, Hooft L. Reporting quality of diagnostic accuracy studies: a systematic review and meta-analysis of investigations on adherence to STARD. Evid Based Med. 2014 4;19(2):47–54. 10.1136/eb-2013-101637 24368333

[40] Cochrane Database of Systematic Reviews. London: John Wiley &Sons, Inc.; 2017. Available from: http://www.cochranelibrary.com/cochrane-database-of-systematic-reviews/ [cited 2017 June 9].

[41] Specifications and quantities for efficient procurement of essential equipment and laboratory commodities for HIV. Geneva: World Health Organization; 2014. Available from: http://apps.who.int/iris/bitstream/10665/103311/1/9789241506519_eng.pdf [cited 2017 June 9].

[42] Recommended selection criteria for procurement of malaria rapid diagnostic tests. Geneva: World Health Organization; 2016. Available from: http://www.who.int/malaria/publications/atoz/rdt-selection-criteria.pdf?ua=1 [cited 2017 June 9].

[43] Ardizzoni E, Fajardo E, Saranchuk P, Casenghi M, Page AL, Varaine F, et al. Implementing the Xpert MTB/RIF® diagnostic test for tuberculosis and rifampicin resistance: outcomes and lessons learned in 18 countries. PLoS One. 2015 12 15;10(12):e0144656. 10.1371/journal.pone.0144656 26670929 PMC4682866

[44] Cost effectiveness and strategic planning (WHO-CHOICE). Geneva: World Health Organization; 2014. Available from: http://www.who.int/choice/en/ [cited 2017 June 9].

[45] Dowdy DW, Cattamanchi A, Steingart KR, Pai M. Is scale-up worth it? Challenges in economic analysis of diagnostic tests for tuberculosis. PLoS Med. 2011 7;8(7):e1001063. 10.1371/journal.pmed.1001063 21814496 PMC3144197

[46] Marseille E, Larson B, Kazi DS, Kahn JG, Rosen S. Thresholds for the cost–effectiveness of interventions: alternative approaches. Bull World Health Organ. 2015 2 1;93(2):118–24. 10.2471/BLT.14.138206 25883405 PMC4339959

[47] Laboratory quality stepwise implementation tool. Geneva: World Health Organization; 2015. Available from: https://extranet.who.int/lqsi [cited 2017 June 9].

[48] Klatser PR. Essential diagnostics–the role of the Department of Biomedical Research of the Royal Tropical Institute in the 21st century. Mem Inst Oswaldo Cruz. 2000;95 Suppl 1:41–2. 10.1590/S0074-02762000000700006 11142722

[49] Houpt ER, Guerrant RL. Technology in global health: the need for essential diagnostics. Lancet. 2008 9 13;372(9642):873–4. 10.1016/S0140-6736(08)61377-X 18790297 PMC2929659

[50] Schroeder LF, Guarner J, Elbireer A, Castle PEAT, Amukele TK. Time for a Model List of Essential Diagnostics. N Engl J Med. 2016 6 30;374(26):2511–4. 10.1056/NEJMp1602825 27355530

[51] Proposal for a WHO model list of essential in vitro diagnostics (or the EDL). Geneva: World Health Organization; 2017. Available from: http://www.who.int/selection_medicines/committees/expert/21/applications/Proposal_for_a_WHOModelListofEssential_In_Vitro_DXs_2017.pdf?ua=1</eref [cited 2017 June 9].

[52] Procedures for product testing and lot testing. Information for RDT manufacturers and procurers. Geneva: World Health Organization; 2015. Available from: http://www.who.int/malaria/publications/atoz/who-find-rdt-procedures-dec-2015.pdf?ua=1 [cited 2017 June 9].

